# Nutritional factors associated with distribution of Mopani Worms in Mopani woodlands in Tsholotsho and Gwanda Districts, Zimbabwe: A comparative survey

**DOI:** 10.1038/s41598-019-53923-7

**Published:** 2019-11-21

**Authors:** Wilfred Njabulo Nunu, Buhlebenkosi Ncube, Oliver Dube, Clever Mpofu, Brighton Ndlovu, Tariro Dzinomwa, Nkosana Khumalo

**Affiliations:** 1 Department of Environmental Science, Faculty of Applied Sciences, National University of Science and Technology, Bulawayo, Zimbabwe; 2 Scientific Agriculture and Environment Development Institute, Bulawayo, Zimbabwe; 3 Department of Chemistry, Faculty of Applied Sciences, National University of Science and Technology, Bulawayo, Zimbabwe; 4 Department of Applied Mathematics and Statistics, Faculty of Science and Technology, Midlands State University, Gweru, Zimbabwe

**Keywords:** Socioeconomic scenarios, Ecosystem ecology

## Abstract

Mopani worms are abundant in Gwanda and sporadic in Tsholotsho though the two areas have similar climatic conditions. The study sought to determine nutritional factors that could be associated with distribution of Mopani worms in these two districts. Ten sampling points in undisturbed Mopani woodlands were established in each district. Samples were collected and analysed in the lab to determine the levels of crude protein, tannin and natural detergent fibres levels in leaves and pH, Nitrates, Phosphates and Potassium levels in soil and Welch’s test, Wilcoxon-Mann-Whitney, Analysis of Variance and the Bonferroni Confidence Intervals were employed to test for significance in the observed differences. Findings showed differences in tree size and leaf length whilst the differences of all other variables (non-extractible tannis, extractible tannis crude protein levels and natural detergent fibres) relating to leaf sample analysis were not statistically significant. Findings on soil sample analysis pointed out that Gwanda had higher pH, Phosphorus and Potassium levels whilst Nitrates were significantly higher in Tsholotsho. Differences in the tree sizes and leaf sizes of the samples from the two sites show that there could be host selection based on these variables.

## Introduction

Mopani Worm harvesting and trade has been identified as a source of income for the rural populaces living in proximity to Mopani woodlands in countries like Botswana, Zimbabwe, South Africa and Namibia^1–3^. The worm is also harvested to supplement diets in these rural areas^4^. Dried Mopani worms are nutritious with protein levels estimated at around 65% complemented with high energy content at about 543Kcal/100 g^4^.

Trading Mopani worms can generate meaningful income in these communities, which can be used to send their children to school or meet their day-to-day living expenses^2,5^. Normally when rains are good, these worms occur twice a year i.e. December – January and April to May^4^. It is estimated that the price of these Mopani Worms range from USD $0.62 to $ 4.00 per kilogram, with the variations depending on the abundance and availability of buyers^2–10^.

Despite similar environmental and climatic conditions Mopani Worms are abundant in Gwanda District compared to Tsholotsho District despite the richness of the host tree (Mopani Tree) in both districts. It is not therefore not well understood why this worm is absent or sporadic in Tsholotsho. This study therefore sought to determine if there are nutritional factors associated with distribution of Mopani worms in these two districts.

## Materials and Methods

### Study areas

**Figure 1:**
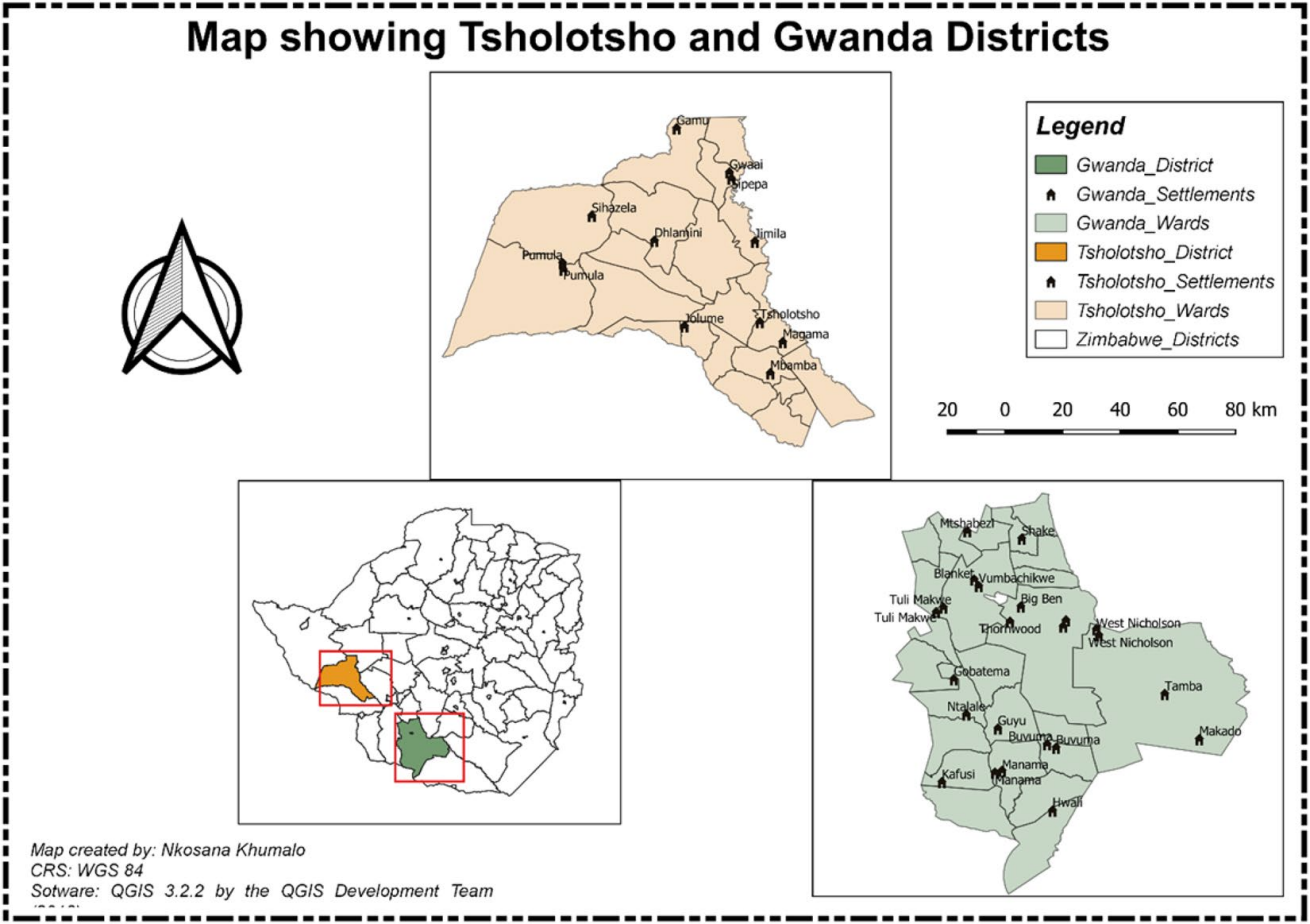
Map showing Tsholotsho and Gwanda Districts.

**Figure 2:**
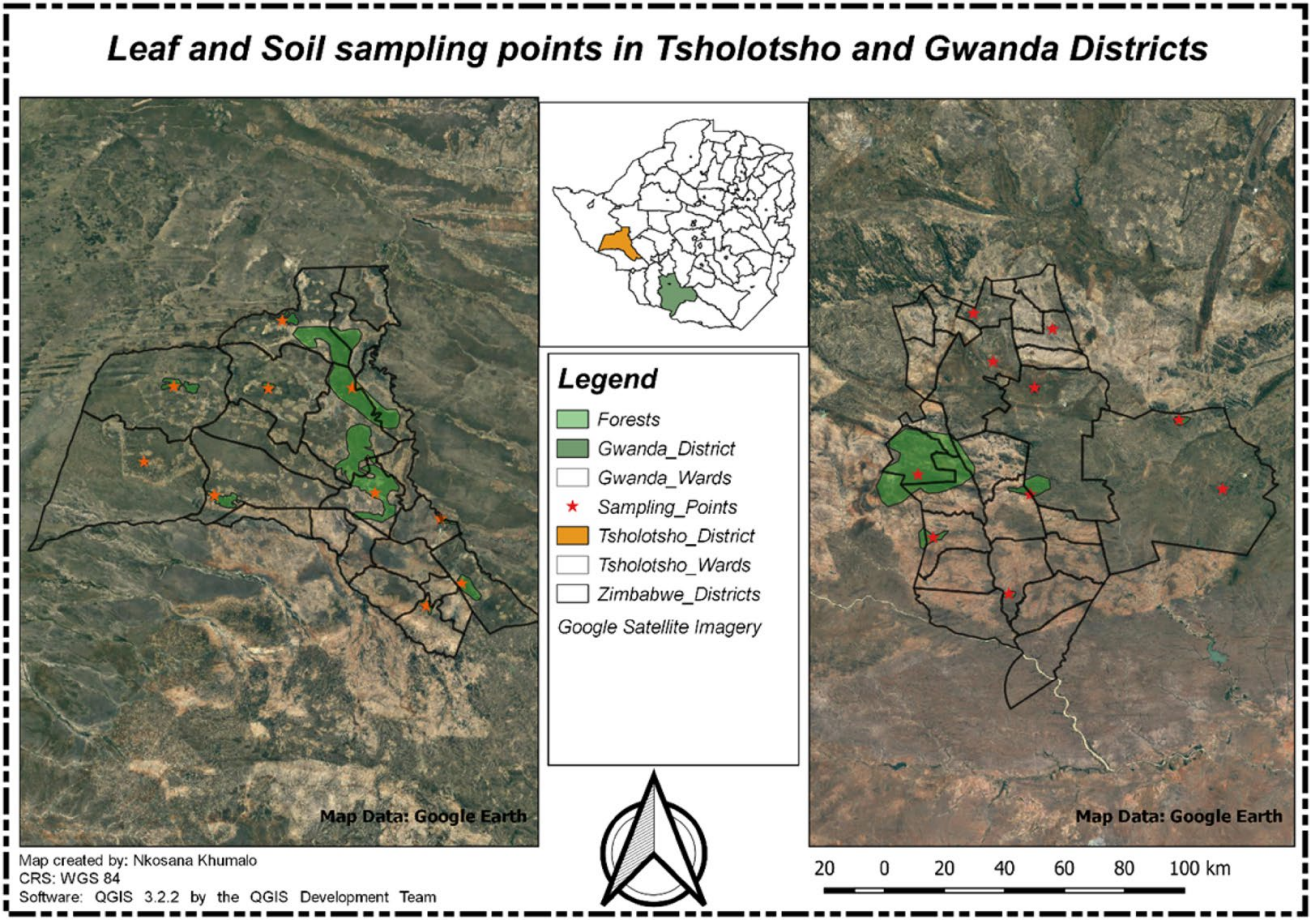
Leaf and Soil sampling points in Tsholotsho and Gwanda Districts.

This research was conducted in two districts that is, Tsholotsho and Gwanda that are both in Zimbabwe. The map of these study areas is presented as Fig. 1. Figures 1 and 2 were generated by one of the authors using QGIS 3.2.2. The compass used to generate the maps was generated automatically as an SVG marker using QGIS. The satellite imagery was downloaded as a Tile Map Service (TMS) and the TMS for Google Earth imagery which is loaded as an XYZ layer in QGIS and has the following URL https://www.google.cn/maps/vt?lyrs=s@189&gl=cn&x=(1)&y=(2)&z=(3) and an attribution text was added on the images in Fig. 2. The two maps denoted as Figs.1 and 2 were then generated using QGIS 3.2.2.

#### Tsholotsho

Tsholotsho district is located approximately 100 km North of Bulawayo in Matabeleland north Province in Zimbabwe^11^. The district is in the region 4-5 which receives low amounts of rainfall of about 350–600 mm^11,12^. The district experiences frequent droughts. Temperatures during summer average around 24 °C whilst in winter it drops to about 15 °C^13,14^. The district lies at an altitude of about 1100 m above sea level. The district is characterised by poor rural populace who practice subsistence farming and also rely on forest natural resources to augment their income^7^. Major activities in this district is timber logging and cattle ranching^4,7,11^.

#### Gwanda

Gwanda district is located approximately 120 km south of Bulawayo in Matabeleland South province^15^. The district lies at an altitude of 1001 m above sea level. The district is also characterised as Region 4–5 which also experiences low amounts of rainfall. Temperatures range from 18 to 30 °C^15^. The annual rainfall received in this district is estimated at between 300 to 700 mm^15,16^. This low amount of rainfall has necessitated construction of water reservoirs to aide survival of food crops^15,16^. Major activities to generate income in this district are: subsistence farming, cattle ranching, brick moulding, gold panning, fishing, vending (including trading in Mopani worms) and cross border trading^15,17,18^.

### Study design

An observational study was conducted and it involved collection and analysis of leaf and soil samples in the Laboratory for variables of interest. This design enabled quantification and comparison of different variables of interest between the two districts.

### Variables

The following variables were considered in this study with regard to Mopani trees and samples collected from the trees: tree size, leaf length, crude protein content in leaves, tannins, and detergent fibres. These variables were chosen because they have been reported to influence feeding practices and distribution of some living organisms that rely on trees for they nutrition^19–21^. Furthermore soils were tested for pH, nitrates, potassium and phosphorus as these variables are associated with tree growth and development thus possible influencing availability of food for the Mopani worms^22^.

### Sample collection, preparation and analysis

A total of ten sampling points per district were identified, these were informed by the fact that the point should be in the Mopani woodlands that were undisturbed during the time of sample collection. This gave a total of twenty sampling points that is, ten in Tsholotsho and ten in Gwanda. The distribution of the sampling points is shown in Fig. 2.

#### Estimating tree size and analysis of leaf samples

A plot measuring 20 m by 20 m was set up in each of the twenty sampling points in the two districts that is, 10 in Tsholotsho and 10 in Gwanda.

Estimating tree size. Five trees were selected from each plot four at the corners of the plots and one in the middle giving a total of 50 trees per district. Tree size was estimated using a method that was adopted from Hrabar *et al*.^23^ and findings presented in cubic metres^23^. An estimation of canopy volume was used a proxy to represent tree size. The formula $V=2\,\pi {r}^{2}h$was used to estimate canopy volume where:

V = canopy volume

*π* = a constant (^22^/_7_)

r = estimated radius as measured length of longest branch off the tree trunk

h = estimated height of the canopy

Collecting leaf samples. Three leaf samples were collected from each of the five trees on the plots giving a total of 15 leaves per plot giving a total of 150 leaf samples per district. Leaf lengths were then measured and averaged per plot and then later on averaged per district. The collected leaf samples were sun dried for two days and ground into powder that would pass through a 1 mm sieve. Three samples of the leaf powder per district were therefore tested for total nitrogen (to determine crude protein levels) using the Kjeldahl method^24^, non-extractible tannins using acidified vanillin as adopted from Broadhurst (1978)^25^ and neutral detergent fibres as adopted from Hall *et al*.^26^. The means of all measured or estimated variables were calculated based on the sample analysis and comparisons within and between the two districts made.

#### Soil samples

One kilogram soil samples were also collected from each sampling plot (i.e. 20 m by 20 m) used for leaf sampling giving a total of 10 samples per district. Soil samples were collected using a soil augur and transferred to containers for transportation. The soil samples were air dried and analysed for pH using the EPA method 9045D as presented by Carnin *et al*.^27^, available nitrates (NO_3_-N) as adopted from Keeney and Nelson (1982)^28^ available phosphorus and exchangeable potassium (K) as adopted from Wolf and Beegle^29^.

### Data analysis

The study adopted the Welch’s test to compare equality of means of tree size and leaf length and all variables associated with soil samples. The Welch’s test is a robust test of equality of means as it caters for type 1 errors and unequal variances and gives a more accurate inference and comparison. The null hypothesis assumed that the two population (from Tsholotsho and Gwanda Districts) means were equal. Therefore we will reject the Null Hypothesis if the absolute value of the t-calculated is greater than the critical value obtained. Furthermore the Wilcoxon-Mann-Whitney test was used to compare the means for crude protein, non-extractible tannins and neutral detergent fibres as these variables were non-parametric. This test is an appropriate for continuous, non-normally distributed data^30^. The null hypothesis in this study was that the means of the two districts were equal, whilst the alternative hypothesis assumes the means are not equal. Therefore the null hypothesis will be rejected if the p-value is less than 0.05. The Bonferroni intervals were also utilised. We rejected the null Hypothesis if the mean difference lies within the specified range of the intervals. We also used the ANOVA to determine whether or not there were significant differences in soil parameters that were analysed within the two districts. Data analysis was conducted on STATA Version 13SE.

### Ethics approval and consent to participate

Permission to conduct the study was sought from the Department of Environmental Science and Health at the National University of Science and Technology.

## Results

### Tree and leaf sizes

Gwanda had significantly larger trees as compared to Tsholotsho. Furthermore leaves in Gwanda are significantly longer than leaves in Tsholotsho. These findings are presented in Table 1.

**Table 1:** Leaf Sample Analysis Results.

Sampling Area and Variable	Mean Value	Welch’s P value	T-value	T-critical	Mann Whitney U Test Value	Mann Whitney U P -Value
**Tree size (m**^**3**^**) (n = 50 in each site)**						
G	169.726	0.022	2.920	2.133		
T	99.392					
**Leaf length (cm) (n = 150 in each site)**						
G	11	0.026	3.124	2.571		
T	7					
**Crude Protein (CP/g DM) (3 aggregated samples Per site)**						
G	13.5				49.5	0.97
T	13.4					
**Non-extractible Tannins (au/g sample) (3 aggregated samples Per site)**						
G	0.14				39	0.123
T	0.08					
**Extractible Tannins (au/g sample) (3 aggregated samples Per site)**						
G	16				23	0.218
T	10.8					
**Natural Detergent Fibres (NDF/g sample) (3 aggregated samples Per site)**						
G	45				45	0.739
T	49					

**Key:** G- Gwanda.

T- Tsholotsho.

### Leaf sample analysis results

The differences in crude protein levels in leaves, non-extractible tannins, extractible tannins and natural detergent fibres in Tsholotsho and Gwanda districts were not statistically significant. These findings are presented in Table 1.

### Soil sample analysis results from the two districts

There were no significant differences on the measured variables within the districts except for exchangeable Potassium in Tsholotsho. There was however significant difference on all variables that were tested on soil samples (i.e. PH, available nitrates, potassium and phosphorus) between the districts. This was further corroborated by the Bonferroni confidence intervals as the calculated mean differences fell within the Bonferroni confidence Intervals. This meant that PH, Phosphorus and Potassium were significantly higher in Gwanda as compared to Tsholotsho, whilst nitrates were higher in Tsholotsho than in Gwanda. These findings are presented in Table 2.

**Table 2:** Soil Sample analysis results for the two study areas.

Sample	pH	Available N_3_0-N	Available P (Mg/Kg)	Exchangeable K (Mg/Kg)
Site	H_2_0 Extraction	1 M KCl Extraction	MEHLICH3 Extraction	MEHLICH3 Extraction
TSP 1	6.47	1.4	4.59	114
TSP 2	6.27	1.57	4.44	115
TSP 3	6.6	0.97	4.48	122
TSP 4	6.34	1.36	4.49	112
TSP 5	6.55	1.43	3.99	125
TSP 6	6.46	1.34	4.39	114
TSP 7	6.47	1.57	4.37	113
TSP 8	6.32	1.4	4.62	156
TSP 9	6.5	1.27	4.39	117
TSP 10	6.23	1.55	4.66	130
Comparison within the District (ANOVA)	0366	0.160	0.175	0.022
GSP 1	6.53	0.13	4.39	159
GSP 2	7.07	0.26	4.66	145
GSP 3	6.85	0.26	4.69	140
GSP 4	7.32	0.4	4.67	160
GSP 5	6.92	0.14	4.63	146
GSP 6	7.12	0.27	4.62	145
GSP 7	7.07	0.27	4.66	138
GSP 8	6.85	0.13	4.66	152
GSP 9	7.12	0.38	4.67	161
GSP 10	7.12	0.47	4.39	150
Comparison within the District (ANOVA)	0.053	0.387	0.470	0.462
Mean Difference	0.576	−1.115	0.162	27.8
Bonferroni Confidence Intervals	0.410–0.742	−1.257–0.973	0.015–0.309	17.352–38.248
Welch’s P - Value	0.000	0.000	0.0347	0.000
Calculated value	−7.270	16.489	−2.523	−5.590
Critical value	2.145	2.120	2.131	2.131

**TSP**- Tsholotsho Sampling Point: **GSP**- Gwanda Sampling Point.

## Discussion

Gwanda had significantly larger trees as compared to Tsholotsho. We would have expected to find significantly larger trees in Tsholotsho and high density of Mopani worms as well as compared to Gwanda as the levels of nitrogen were higher. A study conducted by Lynch et al. (1954) found a direct correlation between tree size and the amount of nitrogen where places with higher levels of nitrogen having larger trees^31^.Their study further reports that high presence of phosphorus and potassium impacts on the intensity of greening in leaves and does not impact much on tree size. Therefore nitrogen as found in nitrates has the most direct effect on yield and tree size^31^.

The study found that there were differences in leaf length in the two districts. This finding contradicts what Hrabar *et al*.^23^ who did not find an association between leaf length and preferences of Mopani worms^23^. However in the current study it was found that Mopani worms were abundance in the district where leaf lengths were significantly longer and this shows an association which could explain the abundance of Mopani worms in Gwanda as compared to Tsholotsho.

“The differences of tannin levels between the two districts” were not statistically significant. Tannins have been proven to be deterrents in browsers^31,32^. Our findings are confirmed by a study by Hrabar *et al*. in 2009 on tree host preference that also did not find any association between tannin levels as an influencing factor in host tree preference^23^. The study also did not find any relationship between crude protein levels, natural detergent fibres and the distribution of Mopani worms. This contradicts a study by Robbins *et al*.^33^ that found a relationship between tannin levels and crude protein i.e. the higher the tannin levels the lower the crude protein that is found in leaves^33^. This then would have meant that protein levels in leaves from Gwanda should have been lower than those in Tsholotsho because the tannin leaves were higher in Gwanda. This was not the case as this study did not find any differences in crude protein levels from the samples from the two districts.

With regards to soil, Gwanda district had significantly higher pH, Phosphorus, and Potassium. These nutrients are necessary for host tree growth and development^34^. Some authors have found an association between tree size at habitat level and host preference^23^. The bigger the trees the higher the chances of finding Mopani worms taking habitat there^23^.

Nitrates were significantly higher in Tsholotsho as compared to Gwanda. It should be noted that nitrates have been associated with high protein levels in leaves^35^. We would therefore had expected to find high levels of crude proteins in Tsholotsho. This was therefore not case as the crude protein differences were not statically significant.

### Limitations of the study

It should be noted that leaf samples were all mixed and ground together per district and only 3 samples analysed per district in the Lab due to financial constraints as this study was not funded.

## Conclusions

Mopani worms were abundant in Gwanda where the tree sizes were bigger and the leaf lengths were longer. Findings from this study suggest that if nutritional factors (as determined by amount of crude protein in leaves) would be an influencing factor in distribution of Mopani worms, we would have expected to find the worm in abundance in Tsholotsho as well. This then provides a window of opportunity to conduct more studies on other potential factors that could influence the distribution of Mopani worm butterflies that could have their own preferences. This would in turn enable for more diverse findings that could help explain the findings of the current study.
